# Vitreomacular Interface Disorders in Behçet’s Uveitis

**DOI:** 10.4274/tjo.77632

**Published:** 2017-10-27

**Authors:** Hilal Eser Öztürk, Özlem Eşki Yücel, Yüksel Süllü

**Affiliations:** 1 Ondokuz Mayıs University Faculty of Medicine, Department of Ophthalmology, Samsun, Turkey

**Keywords:** Behçet’s uveitis, vitreomacular interface, optical coherence tomography

## Abstract

**Objectives:**

To determine the prevalence of vitreomacular interface (VMI) disorders in patients with Behçet’s uveitis and assess the relationship between VMI disorders and clinical characteristics.

**Materials and Methods:**

The macular optical coherence tomography (OCT) images of 160 eyes of 96 patients with Behçet’s uveitis who were being followed in the Uvea-Behçet’s clinic were assessed retrospectively for VMI disorders including posterior vitreous detachment (PVD), epiretinal membrane (ERM), vitreomacular adhesion (VMA), vitreomacular traction (VMT), full-thickness macular hole (FTMH), lamellar hole (LH) and pseudohole.

**Results:**

Twenty-four patients (25%) with unilateral and 72 patients with bilateral uveitis were included in the study. Six (30%) of 20 eyes with anterior uveitis and 57 (40.7%) of 140 eyes with posterior uveitis, in total 63 (39.4%) eyes of 160 eyes had at least one VMI disorder. PVD was detected in 4 eyes (2.5%), ERM in 48 eyes (30%), VMA in 12 eyes (7.5%), and LH in 1 eye (0.6%). None of the eyes had VMT, FTMH, or pseudohole. ERM was detected in 13 eyes (8.1%) on fundus examination and 48 (30%) eyes by OCT (p=0.001). VMI was detected in 12 (50%) of 24 eyes with prior intraocular surgery and 51 (37.5%) of 136 eyes without. The mean duration of uveitis was 7.3±5.8 years in patients with VMI disorders and 5.8±7.7 years in patients without (p=0.04). There was no relation between VMI disorders and anatomic location of uveitis, history of past ocular surgery, number of ocular/periocular steroid injections, or visual acuity.

**Conclusion:**

VMI disorders are common in patients with Behçet’s uveitis. Their frequency increases with the duration of uveitis. OCT is more sensitive than fundus examination in the detection of VMI disorders.

## INTRODUCTION

The vitreous is responsible for globe stabilization, and its relationship to the retinal surface is extremely complex. With aging, the vitreous starts to separate from the retina due to vitreous liquefaction and the weakening of vitreoretinal connections. This separation culminates in total posterior vitreous detachment (PVD) unless it is complicated by one of the vitreomacular interface (VMI) pathologies such as epiretinal membrane (ERM), vitreomacular adhesion (VMA), vitreomacular traction (VMT), full-thickness macular hole (FTMH), lamellar macular hole (LMH), or pseudohole. These pathologies can either occur idiopathically with advancing age or be triggered by intraocular inflammation such as uveitis. They may be completely asymptomatic or cause vision disorders including decreased visual acuity, photopsia, or metamorphopsia.^1,2^ Moreover, they have been shown to impact treatment success when treating complications such as uveitic macular edema.^3^

Optical coherence tomography (OCT) is considered the gold standard in identifying these pathologies, determining prognosis, and in treatment and follow-up.^2,4,5^

The aim of this study was to evaluate the frequency of VMI pathologies in patients with Behçet’s uveitis as measured by OCT and determine their relation with uveitis duration and clinical findings.

## MATERIALS AND METHODS

The study included 160 eyes of 96 Behçet’s uveitis patients, comprising 26 females (27.1%) and 70 males (72.9%), who were followed in the Uvea-Behçet’s unit of the Ophthalmology Department of Ondokuz Mayıs University Faculty of Medicine between March 2015 and June 2016. Approval for the study was obtained from the Clinical Research Ethics Committee of Ondokuz Mayıs University and the Declaration of Helsinki was observed.

Data from the patients’ most recent examination and OCT images obtained on the same date were reviewed retrospectively. Eyes that could not be imaged with OCT or yielded images with a signal strength of 6/10 or below were excluded from the study. The patients’ age, duration of uveitis, site of involvement, presence of cataract, and any previous treatments and intraocular surgeries were recorded.

The Cirrus 512x128 macular cube measurements of all patients obtained by Cirrus Spectral Domain OCT (Carl Zeiss Meditec, Jena, Germany) were reviewed. The images were evaluated with regard to the presence of VMI pathologies such as PVD, ERM, VMT, VMA, FTMH, LMH, and pseudohole. The classification criteria of the International Vitreomacular Traction Study Group were used for defining these VMI pathologies (Table 1).^2^ The definitions are summarized in Table 1. Macular thickness measurements were recorded for all patients.

We assessed the correlations between presence of VMI pathologies and age, duration and location of uveitis, average best corrected visual acuity, lens status, average number of posterior sub-Tenon’s steroid injections, number of intraocular steroid injections, and clinical findings such as macular thickness. The correlation between those same parameters and ERM, one of the interface pathologies, was analyzed separately. Data related to the interface pathologies identified by OCT were used in the statistical analyses. In addition, we determined the extent to which PVD and ERM can be identified by fundus examination and/or OCT.

### Statistical Analysis

The data obtained during the research was analysed using SPSS version 21.0 (SPSS, Chicago, IL, USA) software package. T-test was used in intergroup comparisons of continuous quantitative data with normal distribution; Mann-Whitney U test was used for comparisons of those that did not have normal distribution. Chi-square test was used in comparisons of discrete data. P values below 0.05 were accepted as statistically significant.

## RESULTS

The analysis included the medical records and macular OCT data of 96 patients with Behçet’s uveitis, 12 (12.5%) with anterior segment involvement and 84 (87.55%) with posterior segment involvement, who presented between March 2015 and June 2016. Uveitic involvement was unilateral in 24 patients (25%) and bilateral in 72 patients (75%).

**Figure 1:**
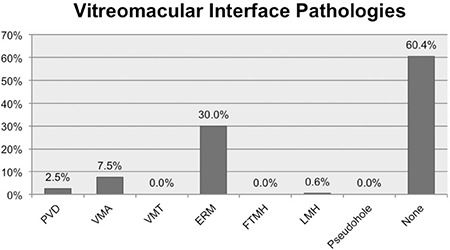
Prevalence of vitreomacular interface disorders detected with optical coherence tomography PVD: Posterior vitreous detachment, VMA: Vitreomacular adhesion, VMT: Vitreomacular traction, ERM: Epiretinal membrane, FTMH: Full-thickness macular hole, LMH: Lamellar macular hole, VMID: Vitreomacular interface disorder

The OCT images of 160 eyes met the inclusion criteria and were evaluated. Sixty-three (39.4%) of the 160 eyes exhibited at least one VMI pathology on OCT (Figure 1). Furthermore, macular edema was detected in 15 eyes (9.4%) and macular atrophy in 41 eyes (25.6%). Although no FTMH was evident on OCT, 3 (3.1%) of the patients had previously undergone surgery for FTMH and achieved anatomic success. Including these 3 eyes, there were a total of 93 eyes (58.1%) with at least one macular pathology.

Assessment of the correlations between VMI pathology and age, duration and location of uveitis, average best corrected visual acuity, lens status, average number of posterior sub-Tenon’s steroid injections, number of intraocular steroid injections, and macular thickness revealed significant correlation only between VMI pathology and duration of uveitis (p=0.045). The other data are shown in Table 2. The correlation between number of attacks per year and the presence of VMI pathology was examined but the result was nonsignificant (p=0.973). The correlation between average number of attacks and the presence of VMI pathology was also evaluated. The average number of attacks was 3.55±3.32 in the VMI pathology group and 3.49±4.56 in the non-VMI pathology group, which was not a statistically significant difference (p=0.107). ERM, which is the most common VMI pathology and has the greatest effect on vision, showed statistically significant correlations with duration of uveitis (p=0.041), visual acuity (p=0.009), and previous cataract surgery (p=0.005). The other data are shown in Table 3. While number of attacks per year was not significantly correlated with the presence of ERM (p=0.745), the average number of attacks was 4.00±3.6 in the ERM group and 3.30±4.30 in the non-ERM group, which was a statistically significant difference (p=0.008). Although OCT revealed PVD in only 4 (2.5%) of the eyes, the patients’ medical records indicated that PVD was detected in 12 eyes (7.5%) on fundus examination (p=0.071). In contrast, ERM was detected in 13 eyes (8.1%) on fundus examination and 48 eyes (30%) on OCT, which was a statistically significant difference (p<0.05).

## DISCUSSION

While VMI pathologies can adversely affect visual acuity and quality, they may also manifest as clinically asymptomatic conditions detectable only by OCT. In particular, VMA without traction and localized PVD without posterior hyaloid thickening do not cause visual symptoms. However, studies conducted among patients with age-related macular degeneration (AMD) have shown that VMI pathologies strongly impact the efficacy of intravitreal anti-VEGF (vascular endothelial growth factor) therapy.^6,7^ Consequently, Munk et al.^3^ examined the effects of VMI pathology in the treatment of uveitis-related macular edema and demonstrated a larger and faster reduction in central retinal thickness in the PVD group as compared to the group with VMA but no PVD; however, they did not observe significant differences in visual acuity improvement or retinal volume. They reported that the detection of VMI pathology may be important in monitoring the treatment of uveitis complications and determining visual prognosis.

In our evaluation of VMI pathologies in patients with Behçet’s uveitis, we identified at least one of these pathologies in 39.4% of the eyes, with PVD in 4 eyes (2.5%), ERM in 48 (30%), VMA in 12 (7.5%), and LMH in 1 eye (0.6%). Three eyes (3.1%) had previously undergone surgery for FTMH. This is consistent with two major studies in the literature, in which Tugal-Tutkun et al.^8^ reported this rate as 2.6% and Benchekroun et al.^9^ as 3.4%. Munk et al.^3^ reported prevalences of 44.1% for VMA, 40.7% for PVD, and 39% for ERM in patients with uveitic cystoid macular edema. These percentages seem very high compared to the data we obtained in our study. This may be because our study was based on the data of all Behçet’s uveitis patients, with or without complications, while Munk et al.^3^ included only cases complicated by cystoid macular edema. In addition, because we used only OCT data to determine PVD prevalence, some cases of PVD may have been overlooked because they were not within the field of the acquired image. Munk et al.^3^ used clinical examination findings as well as OCT to diagnose PVD. They described the OCT appearance of incomplete PVD as a preretinal, thin, hyperreflective band not connected to the macula. To diagnose complete PVD, they examined the medical records of patients whose vitreous margin was not visible on OCT scans and based their decision on the presence of Weiss ring and other signs of complete PVD in the examination findings. Although we mentioned the number of PVDs we detected during examination, these examination findings were not included in the analysis because the focus of our study was identifying VMI pathologies using OCT. PVD was determined to be the most common interface pathology in a study conducted on patients with neovascular AMD.^7^ However, PVD is seen less frequently than VMA in uveitic patients. Munk et al.^3^ have attributed this to the difference in age between patients in the AMD and uveitis groups. While age-related liquefaction of the vitreous leads to PVD, intraocular inflammation may contribute to the formation of VMA in younger uveitis patients.^3^ There are also differences between age-related and uveitic VMI pathologies with regard to their mechanisms of formation. It was reported that the formation of idiopathic ERM occurs secondary to glial cell migration from the retinal nerve fiber layer, and requires retinal pigment epithelial (RPE) cells.^10^ However, uveitic ERMs are different from idiopathic ERMs in that their mechanism of formation does not involve RPE and they contain a large number of inflammatory cells.^11^

In the present study, we identified at least one VMI pathology, macular edema, or macular atrophy in 65% of the eyes. Liu et al.^12^ found this ratio to be 58.6% in their study on the uveitic population living in China. A particularly remarkable aspect of their study was that the rates of ERM (12.6%) and foveal atrophy (8.9%) were lower than in our study. A possible explanation for this discrepancy is that our study included patients with Behçet’s uveitis, which is a specific uveitis group in which posterior segment involvement is more common.

The present study did not reveal statistically significant correlations between the presence of any of the VMI pathologies and any of the demographic or clinical data except for duration of uveitis. Analysis of the association between VMI pathologies and attack frequency revealed no significant correlation with the patients’ average number of attacks or number of attacks per year. This may be due to our failure to determine the actual attack rate because we could not access patients’ medical records before their presentation and/or because patients may not have visited the hospital for every attack. Although there was no significant association between presence of ERM and number of attacks per year, a correlation was observed between ERM and number of average attacks. We also found that presence of ERM was statistically correlated with longer duration of uveitis and pseudophakia. In addition, mean visual acuity was significantly lower in eyes with ERM compared to eyes without ERM. In their study of the relationship between visual acuity and the morphologic characteristics of ERM, Nazari et al.^13^ reported that central foveal involvement, presence of focal adherence, and foveal internal segment/external segment junction damage were correlated with low visual acuity. They also demonstrated that thick ERMs reduce vision more than thin ERMs, and that the thickness of ERMs is associated with their duration. In an epidemiologic study of uveitic patients, Nicholson et al.^14^ reported that the presence of ERM is correlated with advanced age, duration of uveitis, male gender, cataract surgery history, and intermediate and posterior segment involvement. In our study, 3 (6.3%) of the patients with ERM had anterior segment involvement and 45 (93.8%) had posterior segment involvement. The lack of a statistically significant correlation between the site of involvement and the presence of ERM may be attributable to the low number of anterior uveitis patients.

Our evaluation of the effects of all previous injections and surgical procedures on the development of any of the VMI pathologies and ERM formation specifically revealed no statistically significant correlations with intraocular and posterior sub-Tenon’s steroid injections or surgical procedures other than cataract surgery. Nicholson et al.^14^ compared the eyes of patients with unilateral ERM and reported that a significantly higher proportion of the ERM eyes underwent vitrectomy, retinal laser, and intraocular injection, while there were no significant correlations with the other ophthalmologic surgeries and periocular steroid injections.

Only a third of the ERMs identified on OCT in our study were visible on fundus examination. Other studies comparing fundus photographs and fundoscopic examination with OCT indicated that 37-38% of membranes identified by OCT are overlooked in those methods. Therefore, OCT is currently considered the gold standard in ERM detection.^14,15^ It is nearly impossible to detect localized, shallow PVD or VMA by fundus examination, and it is also difficult to detect VMT and examine macular hole characteristics. OCT plays an important role in the follow-up of VMI pathologies and evaluation of treatment efficacy, as well as determination of visual prognosis.^16^

### Study Limitations

The main limitation of our study was its retrospective design. No data were available regarding patient follow-up prior to their presentation to our clinic.

## CONCLUSION

Identifying the presence of VMI pathologies in uveitic patients can assist in predicting visual prognosis and managing complications. OCT is considered the gold standard in the detection and follow-up of these pathologies. Therefore, it is important to carefully evaluate the VMI in OCT images when following uveitic patients.

## Figures and Tables

**Table 1:**
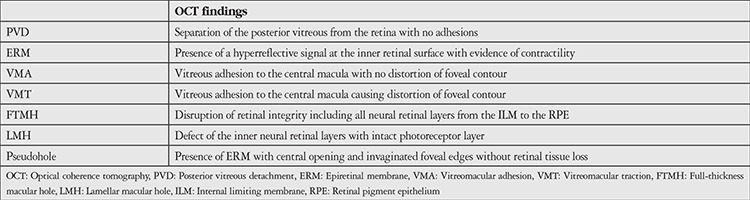
Definitions of vitreomacular interface pathologies

**Table 2:**
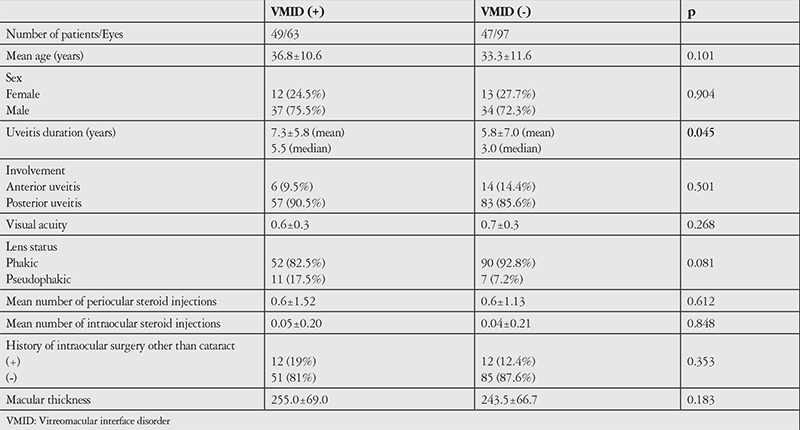
Demographic and clinical characteristics of Behçet’s uveitis patients with and without vitreomacular interface disorders

**Table 3:**
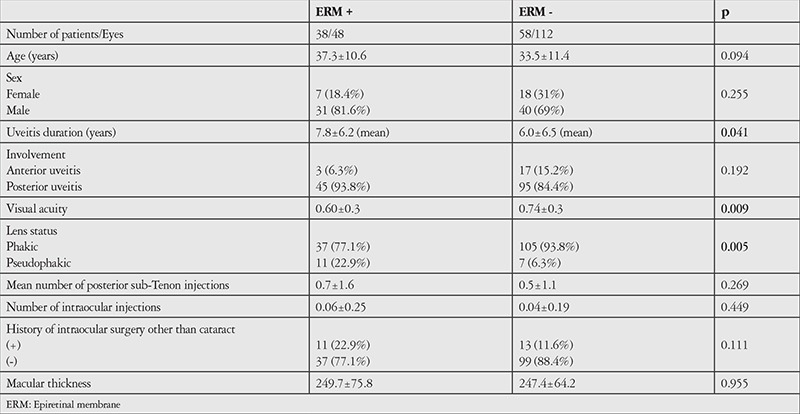
Demographic and clinical characteristics of Behçet’s uveitis patients with and without epiretinal membrane
